# Effect of Platelet-Rich Fibrin Combined With Hyaluronic Acid on Bone Formation in Dental Implant Sockets: An In Vivo Study in Sheep

**DOI:** 10.7759/cureus.64651

**Published:** 2024-07-16

**Authors:** Blend J Ibrahim Almusi, Reiadh K Al-Kamali

**Affiliations:** 1 Department of Oral Surgery, Khanazad Teaching Center, Erbil Health Care Institute, Ministry of Health, Erbil, IRQ; 2 Department of Oral and Maxillofacial Surgery, College of Dentistry, Hawler Medical University, Erbil, IRQ

**Keywords:** advanced platelet-rich fibrin(a-prf), bone formation, hyaluronic acid (ha), dental implants, sheep

## Abstract

Objectives: The goal was to evaluate the effect of the combined growth factor of hyaluronic acid (HA) and advanced platelet-rich fibrin (A-PRF) on acceleration and maturation of bone formation around titanium dental implants in the bone-free space (jumping distance) of an over-preparation socket.

Materials and methods: Thirty-two titanium dental implants were placed in four sheep and distributed into one control group (A) and three experimental groups (B, C, and D) in two different time periods. Each sheep received eight implants. The eight implants in each sheep were distributed into four groups. The first period was one month after the initial placement, 16 implants were used in two sheep. The second period was three months after the initial placement; another 16 implants were used in the other two sheep. All implants were placed in over-prepared implant sockets, resulting in minimal primary stability. In Group A: the space between the dental implant and the bone of the inner wall of the socket was left without a growth substrate material. In Group B: we added HA between the dental implant and the bone of the inner wall of the socket. In Group C: we added A-PRF between the dental implant and the bone of the inner wall of the socket. In Group D: we added a combination of HA and A-PRF between the dental implant and the bone of the inner wall of the socket. Data was collected for each group at one month and three months at the same time. A high-resolution, desktop micro-CT system (Bruker Skyscan 1275, Kontich, Belgium) was used to scan the specimens. The NRecon software (ver. 1.6.10.4, SkyScan) and CTAn (SkyScan) were used for the visualization and quantitative measurement of the samples. One-way analysis of variance (ANOVA) was used to compare the means of the four study groups in the same period. A post hoc test was used after ANOVA to compare the means of two samples at the same time. A p-value of ≤ 0.05 was considered statistically significant.

Results: After one month and three months of using combined HA and A-PRF on Group D, significant acceleration was observed in bone formation in all tests around dental implants compared with other groups, while no significant acceleration was observed when they were used separately; all three study groups showed significant results when compared with the control group.

Conclusion: Our data showed that using a combination of HA and A-PRF had a significant effect on the acceleration of the bone formation and ossification process when added to bone-free space (jumping distance) around implants while leaving space without any growth substrates might delay the bone ossification process.

## Introduction

Dental implants are the most accepted treatment option for the replacement of missing teeth [1]. Successful dental implant therapy depends upon various factors such as patient-related factors as well as dental implant-related factors [2]. Bone ossification, or the process of new bone formation, plays a crucial role in the long-term success of dental implants. Successful osseointegration, where the implant integrates with the bone, is essential for implant stability and longevity [3]. Several factors influence this process, including the biological and structural properties of the bone surrounding the implant, the surface characteristics of the implant, and the molecular signaling pathways involved in bone remodeling and formation [2,3].

Immediate placement of implants has taken the forestage in recent times due to its numerous advantages, such as preservation of alveolar bone, better implant orientation, esthetics, and psychosocial benefits provided to the patients [1-3]. However, one of the drawbacks of using an immediate implant is that there is a space between the implant body and socket wall which cannot be avoided because they are different in size. However, this space is referred to as jump distance and it is located toward the coronal end of the implant. When the jumping distance becomes too long bone resorption and bony defects may occur which reduces the stability of an implant [1,4]. To ensure the success of the immediate implant, this jumping distance must be filled. The use of bone substitutes between the titanium implant surface and the interior walls of the sockets can improve the primary stability of immediately placed implants, biological fixation, and then the osseointegration process [5]. Multiple techniques have been recommended to enhance the osseointegration process. According to experimental research, adding molecules or growth factors to the implant surface may increase osteoblastic activity and improve the functional integration of implants. Beneficial growth factors could be delivered to the surface of implants and neighboring bones using platelet derivatives collected from the patient’s blood [6,7]. 

Platelet-rich fibrin (PRF) is considered a healing biomaterial. It has a robust stimulating effect on various aspects of soft and osseous tissue healing, including angiogenesis, immune control, and the harnessing of circulating stem cells [8,9]. It permits rapid angiogenesis and an easier remodeling of fibrin. The leukocytes and key immune cytokines, such as interleukins 6, interleukins, and tumor necrosis factor α (TNF α) trapped in PRF, give it an anti-infectious effect and let PRF act as an immune regulation mode [10-12]. It features all the necessary parameters permitting optimal healing. The PRF matrix can release various growth factors and cytokines locally at the wound site for a prolonged time, with an important role in various stages of wound healing, promoting periapical tissue generation.

The biological and clinical characteristics of second-generation platelet concentrates, such as advanced platelet-rich fibrin (A-PRF) and leukocyte-platelet-rich fibrin (L-PRF), make them highly promising for use in regenerative medicine. Over the past few decades, they have been primarily utilized in dentistry to enhance the revascularization of damaged tissues and bone regeneration prior to implant placement [13]. 

The PRF matrix especially the second generation of PRF (like A-PRF) has the capability to gradually release a variety of growth factors and cytokines directly at the wound site over an extended period. These substances are crucial in different phases of wound healing, thereby fostering the regeneration of bone tissue [13-15]. A-PRF can release growth factors like bone morphogenetic proteins (BMPs), transforming growth factor-beta (TGF-β) which stimulates the differentiation of mesenchymal stem cells into osteoblasts, vascular endothelial growth factor (VEGF) which enhances angiogenesis [9-15]. Additionally, PRF is used to fill post-extraction sockets to maintain the alveolar bone ridge [13-15] which makes A-PRF a good choice in treating the jumping distance in immediate implant placements. Overall, PRF enhances the healing process around dental implants by providing a rich source of growth factors, supporting cellular activities, reducing inflammation, promoting vascularization, and facilitating new bone formation. These combined effects lead to improved osseointegration and stability of dental implants [5-9,13-15].

On the other hand, hyaluronic acid (HA) is another option to fill the jumping distance between the implant surface and surrounding bone in immediate implant placement. HA is a naturally occurring non-sulfated glycosaminoglycan with a high molecular weight [16]. It is a key element in the soft periodontal tissue, gingiva, and ligament, and in the hard tissues, such as alveolar bone and cementum [17]. Because HA is a crucial molecule in inflammation, epithelium formation, and tissue remodeling, it can regulate the inflammatory response, HA produced by hyaluronan synthase enzymes in periodontal tissues, gingiva, periodontal ligaments, and alveolar bone. Additionally, HA prevents the breakdown of extracellular matrix by serine proteinases generated during the healing phase from inflammatory cells, thus reducing inflammation and stabilizing the granulation tissue [17,18]. It also participates in many biological responses, including angiogenesis, inflammation, wound healing, and bone regeneration [19,20]. HA with different molecular weights might have different biological functions during the process of bone regeneration [21]. According to Zhao et al., HA with low molecular weight could enhance the proliferation of bone marrow-derived mesenchymal stem cells (BMSCs), whereas HA with high molecular weight could enhance mRNA expressions of osteogenic gene markers, such as alkaline phosphatase, Runt-related transcription factor 2 and osteocalcin [22]. Additionally, due to its good physicochemical properties, HA can be used as a vehicle for osteogenesis-related cells and factors for bone repair. Bone is a complex heterogeneous and vascularized tissue with vascular networks, being connected to the blood system by transverse channels [23]. HA and PRF are two injectable agents used in various medical procedures. Xu et al. compared the efficacy of intra-articular injections of HA, platelet-rich plasma (PRP), and PRF for treating temporomandibular disorders (TMDs). According to the study, PRP and PRF exhibited similar short-term efficacy in treating TMDs, while PRF was more advantageous in terms of long-term efficacy [24]. Another study evaluated the outcomes of the intra-socket application of 1% HA oral gel and A-PRF on the expected postoperative complications, pain, swelling, and trismus following the surgical extraction of the impacted mandibular third molar. The study concluded that both HA and A-PRF were effective in reducing postoperative complications; however, A-PRF was more effective in reducing pain and swelling [25]. Based on these studies, it can be inferred that HA and A-PRF are effective agents for reducing postoperative complications; they also have a good effect on the enhancement the bone healing and ossification. However, more investigation is recommended about the optimal concentration and different methods of delivery of HA and PRF to promote ossification in immediate implant placement. 

## Materials and methods

Our prospective, experimental study was performed on four animals (sheep) after obtaining ethical approval from the Ethical Review Committee of the College of Dentistry, Hawler Medical University (Erbil-Iraq) under no: 1023 on 10/06/2021. All the sheep were male, with two to three years old age and about 45 to 55 kg weight. 

Thirty-two titanium dental implants were placed in four sheep and divided into one control group and three experimental groups in two different study time periods. In the first period, one month after the initial placement, 16 implants were used in two sheep. In the second period, three months after the initial placement, another 16 implants were used in the other two sheep. Each sheep received eight implants in the lower posterior border of the mandible with about 4 to 6 mm between them, with four implants on each side of the mandible [26]. The eight implants in each sheep were distributed into four groups: two for the control group (A), and two each for the experimental groups (B, C, and D). The same implant protocol was used in preparing implant sockets. All implant socket was perpetrated to a length of 10mm and a diameter of 4 mm, then a titanium dental implant of size 3.6 mm in diameter and 10 in length (smaller than socket size) was inserted in the preparation socket, acquired minimum implant primary stability and acquired bone free space (jumping distance) between the titanium implant surface and the inner wall of the prepared socket. According to each study group, these spaces were treated with a growth factor material. 

Surgical protocol

First, we anesthetized the sheep by giving them xylazine and ketamine through intra-muscular injection into their legs. The combination of these two drugs to provide sedation and analgesia during the operation, they known to have a wide margin of safety in sheep. The dosage range for xylazine is 0.3 to 0.5 mg/kg, while it is 0.05 to 0.1 mg/kg for ketamine [27]. In this study, local anesthesia lidocaine with epinephrine injection was then used for the lower border of the mandibular jaw. An Idiom 10% solution was used to antisepticise the area. Then a crestal incision was made on the lower border of the mandibular to expose the bone. We started our drilling protocol at 1000 RPM speed with irrigation by normal saline to prepare the implant socket until we reached the diameter (4mm) and length (10 mm) to have more bone-free space, then a smaller-sized dental implant (diameter 3.6 mm: length 7 mm) was inserted into the socket so we got an over-preparation implant socket and minimum implant primary stability and space of bone free between the implant and the inner bone wall. According to each of our study groups, the jumping distance (bone-free space) between the dental implant fixture and the bone wall of the socket was treated. Once all dental implants were inserted, the wound was closed by suturing it with a simple interrupted technique (3-0 Vicryl). After closing the wound, an iodine solution was used to clean the area and prevent infection.

Study groups and implant socket treatment

In this study, we had four groups, one control and three study groups, in which different materials were used to fill the distance between the implant socket wall and implant fixture (jumping distance (bone-free space). Group A (control): In this group, the implant was inserted into the prepared socket without any bone enchantment material; thus, no material was added to the jumping distanced only wound side blood. Group B (HA): In this study group, the implants were inserted into the prepared socket with HA gel. A pure 1% HA gel was used to cover the implant fixture [28] surface and also added to the prepared implant socket; thus, the jumping distance was stuffed with HA. Group C (Advanced platelet-rich fibrin (A-PRF)): In this group, the implants were inserted into the prepared socket with A-PRF type. 20 ml of blood was taken from sheep and put in two glass A-PRF tubes without an anticoagulant agent and then in the Centrifuges (1500rpm for 14 minutes) to make A-PRF [29]. The A-PRF was separated from the other glass tube containing the A-PRF preparation Kit; next, it was added to the implant surface and prepared implant socket to fill the jumping distance. Group D (A-PRF & HA): In this group, the implants were inserted into the prepared socket with a combined material of HA and A-PRF (1:1 ratio). The combined gel material was used to cover the implant fixture surface and the prepared socket in order to fill the jumping distance to see their effect on bone formation around the dental implant.

Study groups and time period

In this study, we had four groups (one control and three study groups) in two different periods; the first result data after one month and the second result data after three months of implant insertion in the bone were obtained. In the first period, 16 implants were distributed to four groups, (four implants per group); after one month of insertion of the implants, the sheep were sent to our animal house care and then painlessly sacrificed and the mandibular bone block with the dental implant (after removing soft tissue) was sent to radiological analysis. In the second period, 16 implants were also distributed to four groups (four implants per group); after three months of insertion of the implants, the sheep were sacrificed, and the mandibular bone block with the implant (after removing soft tissue) was sent to radiological analysis. A high-resolution, desktop micro-CT system (Bruker Skyscan 1275, Kentish, Belgium) was used to scan the specimens.

Postoperative care for sheep

In our study, four sheep were used. The effect of combination materials (HA with A-PRF) is in experimental investigation, and we do not have previous research about their effect on ossification around implants, so we minimize the number of animals in our study [27]. All sheep received identical treatment, attention, and nourishment. The surgical site was cleaned with an iodine solution twice daily for seven days after the operation to ward off infections. Then, meloxicam intra-muscular injection (20 mg/ml) was used to control postoperation pain (per day) for seven days after the operation [30]. Oxytetracycline injection (200 mg) as a broad-spectrum antibiotic was given once per day to prevent postoperative infection for seven days after the operation [31]. All sheep were provided with the same food and care in animal houses for both time periods, and weight was checked every week for weight loss; all considerations were taken by animal house care to minimize animal suffering during the study period. 

Imaging analysis 

The NRecon software (version 1.6.10.4, SkyScan, Kontich, Belgium) and CTAn (version 1.16.1.0, SkyScan) were utilized to visualize and quantitatively analyze the samples. The modified algorithm outlined by Feldkamp et al. was employed to generate axial, two-dimensional (2D), 1000 × 1000-pixel images. Reconstruction parameters included fixing ring artifact correction and smoothing at zero while setting beam artifact correction at 40%. The NRecon software was utilized to reconstruct the images captured by the scanner, resulting in 1023 cross-sectional images; the entire volume was represented in Micro-CT. Additionally, CTAn software was employed for three-dimensional (3D) volumetric visualization, analysis, and area/volume measurements for Micro-CT. All reconstructions were displayed on a 21.3-inch flat-panel color-active matrix TFT medical display (NEC MultiSync MD215MG, Munich, Germany) with a resolution of 2048 × 2560 at 75 Hz and 0.17-mm dot pitch, operating at 11.9 bits. Reconstruction and measurement images were conducted by a dentomaxillofacial radiologist with 18 years of experience (KO).

Following reconstruction, regions of interest (ROIs) measuring 3x3 cm were delineated using CTAn software to encompass both the implant and the surrounding bone throughout the entire specimen. All program specifications were adhered to for the analysis of the microarchitecture in both 2D and 3D formats.

To differentiate resorption cavities from the rest of the specimen, an appropriate threshold was essential. Thus, the threshold was configured as follows: the lower limit ranged between 0 and 255 (in gray values), while the upper one corresponded to the brightest end of the spectrum, representing the highest bone density value. Grayscale images were processed with a Gaussian low-pass filter for noise reduction, followed by automatic segmentation thresholding for the calculation of areas in 2D and volumes in 3D [32]. This process involved transforming the grayscale range into a binary image of black and white pixels. Subsequently, for each slice, an ROI was selected to encompass a single object entirely, enabling volume calculations. Structural parameters, including total tissue volume (TV), bone volume (BV), percent bone volume fraction (BV/TV), bone mineral density (BMD), trabecular thickness (TB.TH), and Density of trabecular connections within a bone sample (connectivity, Conn), were calculated three-dimensionally (3D) based on the ROI volume. Additionally, the BMD of the implant surrounding bone was computed. Skyscan CT-analyzer software (CTAn) facilitated an integrated calibration of datasets into Hounsfield units (HU) and mineral density (MD). Two mineral concentration conical MD phantoms (rods) of 0.25 and 0.75 gHAp cm^3^ were used for appropriate calibration phantom scans and measurements [32,33]. Samples were scanned alongside MD phantom rods placed in an identical tube for calibration purposes. Grayscale values were then converted to mineral density values using a linear calibration curve based on the grayscale values obtained from the two different mineral concentration conical phantoms. Scan parameters, including pixel size, rotation step, frame averaging, voltage, and filter, remained consistent across all scans. To distinguish bone from other structures, a global thresholding method was applied using CTAn software, with the entire procedure being semi-automatic within the same software environment. Grayscale images were processed with a Gaussian low-pass filter for noise reduction, followed by automatic segmentation thresholding for the analysis of bone mineral density in 3D volumes. This involved transforming the grayscale range into a binary image of black and white pixels. Subsequently, for each slice, the same 3x3 ROI was selected for mineral density analysis [32,33].

Statistical analysis

Data were analyzed using IBM SPSS Statistics for Windows, Version 26 (Released 2019; IBM Corp., Armonk, New York, United States). The normality of data was checked using the Shapiro-Wilk test; accordingly, parametric tests were used when indicated. One-way analysis of variance (ANOVA) was used to compare the means of the four study groups. A post hoc test (LSD) was used after ANOVA to compare the means of the two samples. A p-value of ≤ 0.05 was considered statistically significant.

## Results

The present research compared the outcome from each group at two different times. The first time period was one month after treatment; the highest mean of percent bone volume (49.49 mm^3^) was observed in Group D, being significantly higher than the other groups (C, B, and A). The highest BV (1190.42 mm^3^) was also observed in Group D, which was significantly (p < 0.001) higher than that for Groups C (853.59 mm^3^), B (844.35 mm^3^), and A (440.85 mm^3^). There was a significant (p < 0.001) difference between the groups regarding the bone surface. The highest one was in Group D (534.97 mm^2^). No significant difference was detected between the groups in the TV (p = 0.084). However, the difference in tissue surface between the groups was significant (p < 0.001), with the highest mean detected in group D (1244.71 mm^2^). A significant difference was detected between the groups in the trabecular thickness, with the highest mean in Group D (42.91 mm). Teste connectivity, Conn, showed the highest mean (635.25) in Group D, and all the differences between the groups were significant. There was a significant difference between the groups in BMD (p < 0.001), and the highest mean was in Group D (2.88 g/cm) as presented in Table 1.

**Table 1:** Comparing the study variables between the four study groups after one month of treatment *By ANOVA. **By the LSD test. Note: A: Control, B: Hyaluronic acid, C: A-PRF, D: D-Combination between A-PRF and hyaluronic acid A-PRF: Advanced platelet-rich fibrin; BMD: bone mineral density; LSD: least significant difference

	Groups	Mean	SD	P*	Groups	P**
Percent bone volume, BV/TV % (mm^3^)	A	18.60	0.98		A X B	0.003
	B	29.54	3.63		A X C	< 0.001
	C	33.14	4.01	< 0.001	A X D	< 0.001
	D	49.49	6.12		B X C	0.240
	Total	32.69	12.02		B X D	< 0.001
					C X D	< 0.001
Bone volume (mm^3^)	A	440.85	27.74		A X B	< 0.001
	B	844.35	32.27	< 0.001	A X C	< 0.001
	C	853.59	40.74		A X D	< 0.001
	D	1190.42	70.14		B X C	0.780
	Total	832.30	277.31		B X D	< 0.001
					C X D	< 0.001
Bone surface (mm^2^)	A	142.85	36.10		A X B	0.586
	B	163.20	16.18		A X C	0.697
	C	157.36	8.94	< 0.001	A X D	< 0.001
	D	534.97	94.44		B X C	0.875
	Total	249.59	176.43		B X D	< 0.001
					C X D	< 0.001
Tissue volume (mm^3^)	A	2370.83	121.69		A X B	0.022
	B	2896.48	427.84		A X C	0.273
	C	2601.08	304.83	0.084	A X D	0.805
	D	2421.43	172.86		B X C	0.166
	Total	2572.45	330.56		B X D	0.035
					C X D	0.387
Tissue surface (mm^2^)	A	372.10	116.06		A X B	0.776
	B	347.61	29.63	< 0.001	A X C	0.567
	C	322.67	13.29		A X D	< 0.001
	D	1244.71	204.75		B X C	0.772
	Total	571.77	415.48		B X D	0.000
					C X D	< 0.001
Trabecular thickness, SD(Tb.Th) (mm)	A	10.93	0.53		A X B	0.000
	B	16.37	1.61		A X C	0.022
	C	13.91	2.24	< 0.001	A X D	0.000
	D	42.91	1.53		B X C	0.050
	Total	21.03	13.28		B X D	< 0.001
					C X D	< 0.001
Connectivity, Conn	A	59.25	17.27		A X B	< 0.001
	B	387.50	42.59		A X C	< 0.001
	C	264.25	28.39	< 0.001	A X D	< 0.001
	D	635.25	84.19		B X C	0.005
	Total	336.56	219.97		B X D	< 0.001
					C X D	< 0.001
BMD (g/cm)	A	2.46	0.02		A X B	0.002
	B	2.59	0.07		A X C	0.006
	C	2.57	0.03	< 0.001	A X D	< 0.001
	D	2.88	0.05		B X C	0.592
	Total	2.63	0.17		B X D	< 0.001
					C X D	< 0.001

The second time was three months after treatment. There were significant differences between the groups regarding all the studied variables except for intersection surface (p = 0.561), and the mean of the significant variables (mentioned above) in Group D was the highest compared with the others.

Starting from the percent bone volume, the highest mean was in Group D (40.16 mm^3^), C (29.53 mm^3^), B (35.18 mm^3^), and A (20.83 mm^3^), respectively. All the differences between each of the two groups were significant. Nearly the same pattern was followed by bone volume, except that the means were as Group D, B, C, and A, respectively. Bone surface means followed the same pattern as bone volume means, and all the differences between each of the two groups were significant. TV means followed the same pattern of bone surface, and all the differences were significant except for the difference between the means of groups B and C (p = 0.326). The tissue surface followed the same pattern of TV; all the differences were significant except for the difference between the means of Groups B and C (p = 0.104). The means of the trabecular thickness were ranked from the highest to the smallest as follows: means of Groups D, B, C, and A. All the differences between each of the two groups were significant except for Groups B and D (p = 0.523) and Groups B and C (p =0.013). Teste Connectivity, Conn, (density of trabecular connections within a bone) showed the highest mean (726.5) in Group D, and all the differences between the groups were significant.

BMD showed the highest mean (3.05) in Group D, and the differences between Group D and other groups were significant except for Groups B and D (p = 0.075) as presented in Table 2.

**Table 2:** Comparing the study variables between the four study groups after three months of treatment *By ANOVA. **By the LSD test. Note: A: Control, B: Hyaluronic acid, C: A-PRF, D: D-Combination between A-PRF and hyaluronic acid A-PRF: Advanced platelet-rich fibrin; BV: bone volume; TV: tissue volume; BMD: bone mineral density; LSD: least significant difference

	Groups	Mean	SD	P*	Groups	P**
Percent bone volume, BV/TV % (mm^3^)	A	20.83	1.33		A X B	< 0.001
	B	35.18	2.66		A X C	0.002
	C	29.53	0.66	< 0.001	A X D	< 0.001
	D	40.16	5.27		B X C	0.022
	Total	31.42	7.90		B X D	0.039
					C X D	< 0.001
Bone volume (mm^3^)	A	506.12	15.73		A X B	< 0.001
	B	1056.53	90.94		A X C	< 0.001
	C	846.45	23.76	< 0.001	A X D	< 0.001
	D	1319.25	46.33		B X C	< 0.001
	Total	932.09	310.95		B X D	< 0.001
					C X D	< 0.001
Bone surface (mm^2^)	A	284.97	7.78		A X B	< 0.001
	B	447.52	9.97		A X C	0.001
	C	383.96	5.39	< 0.001	A X D	< 0.001
	D	590.85	66.26		B X C	0.021
	Total	426.83	118.58		B X D	< 0.001
					C X D	< 0.001
Tissue volume (mm^3^)	A	2434.43	96.74		A X B	0.001
	B	3001.30	34.50	< 0.001	A X C	0.006
	C	2867.05	78.66		A X D	< 0.001
	D	3319.08	347.77		B X C	0.326
	Total	2905.46	367.65		B X D	0.032
					C X D	0.005
Tissue surface (mm^2^)	A	881.68	72.82		A X B	< 0.001
	B	1395.65	46.32		A X C	< 0.001
	C	1310.89	70.26	< 0.001	A X D	< 0.001
	D	1669.15	78.46		B X C	0.104
	Total	1314.34	298.27		B X D	< 0.001
					C X D	< 0.001
Trabecular thickness, SD(Tb.Th) (mm)	A	30.12	4.71		A X B	< 0.001
	B	42.79	1.62		A X C	0.007
	C	36.77	1.67	< 0.001	A X D	< 0.001
	D	44.15	2.60		B X C	0.013
	Total	38.46	6.31		B X D	0.523
					C X D	0.004
Connectivity, Conn	A	382.00	39.86		A X B	< 0.001
	B	600.25	10.24		A X C	0.002
	C	487.75	7.46	< 0.001	A X D	< 0.001
	D	726.50	60.82		B X C	0.001
	Total	549.13	136.49		B X D	< 0.001
					C X D	< 0.001
BMD (g/cm)	A	2.66	0.14		A X B	0.005
	B	2.91	0.01		A X C	0.142
	C	2.77	0.02	0.001	A X D	< 0.001
	D	3.05	0.15		B X C	0.080
	Total	2.85	0.18		B X D	0.075
					C X D	0.002

## Discussion

Assessing bone quality, quantity, and facial cortical bone thickness is essential for successful immediate dental implant placement [5]. To ensure the success of the immediate implant, we must fill this jumping distance. The use of bone growth enhancement substitutes between the titanium implant surface and the interior walls of the sockets can improve the primary stability of immediately placed implants, as well as accelerate bone formation and the osseointegration process. Using a high-resolution micro-CT system our study gives us fast and accurate reading data about bone formation and maturation around the titanium dental implant because high-resolution micro-CT is a powerful tool for investigating skeletal development, growth, and repair of bone and understanding bone-related processes. Micro-CT imaging of craniofacial bone has facilitated quantitative 3D measurements of trabecular bone morphology parameters, including trabecular thickness (Tb. Th), percent bone volume, BV/TV %, BV, total TV and also measuring BMD [34,35]. In our research, we added different materials (growth factors) to the bone-free space between the dental implant and the inner wall of the socket to enhance the osseointegration process, accelerate bone formation, and get mature bone faster. Adding a new material of HA and A-PRF in combination together to the jumping distance had a significant effect on percent bone volume, bone volume, bone surface, tissue surface, trabecular thickness, connectivity, conn, and BMD after one month and three months as presented in Table 1 and Table 2. This is mostly due to the dual properties of the two materials when mixed, accelerating the process of bone formation. This is consistent with research done on their effect on TMDs by Xu et al. [24], in which a combination of HA and A-PRF showed better results in the healing process of temporomandibular disorders.

The present research data shows that using HA and A-PRF in combination together inside the dental implant socket had shown a better effect on accelerating bone healing processes around the titanium dental implant. The combination of materials had a dual effect on permitting rapid angiogenesis, the proliferation of mesenchymal cells, and increasing osteoblast activities. The PRF matrix especially the second generation of PRF (like A-PRF) can gradually release a variety of growth factors and cytokines directly at the wound site over an extended period. These substances are crucial in different phases of wound healing, thereby fostering the regeneration of bone tissue [13-15]. A-PRF can release growth factors like BMPs, transforming growth factor-Beta (TGF-β) which stimulates the differentiation of mesenchymal stem cells into osteoblasts, and vascular endothelial growth factor (VEGF) which enhances angiogenesis [9-15] in agreement with research done by Shetye et al. [9] and Gupta et al. [14] and many other research studies on the role of PRF in treating and enhancing bone formation [10,12,15]. HA can enhance the mineralization process and work as a scaffold to increase growth factors' effects on bone regeneration. HA has been found to boost the proliferation of BMSCs [21,22]. HA has many physiological functions within the bone and gingival tissues. Also, it can play a regulatory role in the inflammatory response [17], which agrees with many research studies like Zhai et al. [21] and Zhao et al. [22] in enhancing bone healing and formation. Therefore, the primary reason for achieving superior results in the first and second study periods was the dual specifications of the combination materials, as opposed to using them individually within the implant socket. The present research data shows also the HA group also had better results in some tests compared with the A-PRF group in enhancing the osseointegration process in bone volume after three months and teste connectivity, Conn after both study periods (Tables 1, 2). A similar result was found by Abaza et al. [36] while comparing the effect of HA and injectable platelet-rich fibrin (I-PRF) on xenografts in bone defect areas, in which the former showed better results.

 While comparing between HA and A-PRF groups showed no significant result in all micro-CT tests for surrounding bone after one month of the treatment (first time period), and most of the tests after three months (second time period) expect bone volume, connectivity, and conn (Tables 1, 2). This was probably due to their similar effect on enhancing the osseointegration process, healing of bone, and general healing process of the wound. The same result was found by Faour et al. [37] in 2022, in which these two materials were used separately; no significant result was found about their effect on gingival tissue. Both the A-PRF and HA groups showed significant results in micro-CT tests when used separately compared with the control group (their effect on bone formation and maturation) as shown in much research [6-9,21,22]. Therefore, further research is necessary to compare the outcomes of using the two materials separately and in combination, with varying concentrations and application methods, and their impact on the bone formation process. A larger sample size is also required to obtain more reliable results and to evaluate the effects of the combined materials on human bone.

Regarding the limitation of the present study, we need to use a bigger sample size for study groups. It is better to do more study on the same subject with also different period time. we have loss of some growth substrate material when added to the implant socket especially when we have excessive bleeding from a socket. Radiographic analysis is not so accurate to measure osseointegration because of the effect of implant metal on contact surrounding bone images so we need histological slide preparation of samples to measure osseointegration better.

## Conclusions

The present research generally showed that using growth substrate materials, such as HA and A-PRF, had a better result in the healing process of bone, especially when used together. In other words, the combination of these two materials showed better results in bone formation and maturation than being used separately. Leaving the jumping distance with no growth substrates could lead to a delay in bone formation, failing dental implant prosses. We need a bigger sample size, as well as using them on humans to get comprehensive results about their effect on bone formation. Also, other types of second-generation PRF should be used.
